# Optical Solitons with Beta and M-Truncated Derivatives in Nonlinear Negative-Index Materials with Bohm Potential

**DOI:** 10.3390/ma14185335

**Published:** 2021-09-16

**Authors:** Muhammad Bilal Riaz, Jan Awrejcewicz, Adil Jhangeer

**Affiliations:** 1 Department of Automation, Biomechanics and Mechatronics, Lodz University of Technology, 1/15 Stefanowskiego St., 90-924 Lodz, Poland; jan.awrejcewicz@p.lodz.pl; 2 Department of Mathematics, University of Management and Technology, Lahore 54770, Pakistan; 3 Department of Mathematics, Namal University, Talagang Road, Mianwali 42250, Pakistan; adil.jhangeer@namal.edu.pk

**Keywords:** optical solitons, new extended direct algebraic method, graphical structure, bohm potential, negative-index materials

## Abstract

In this article, we explore solitary wave structures in nonlinear negative-index materials with beta and M-truncated fractional derivatives with the existence of a Bohm potential. The consideration of Bohm potential produced quantum phase behavior in electromagnetic waves. The applied technique is the New extended algebraic method. By use of this approach, acquired solutions convey various types of new families containing dark, dark-singular, dark-bright, and singular solutions of Type 1 and 2. Moreover, the constraint conditions for the presence of the obtained solutions are a side-effect of this technique. Finally, graphical structures are depicted.

## 1. Introduction

The subject of fractional calculus (calculus of integrals and derivatives of any arbitrary real or complex order) has attained great importance over the previous three decades or so, due to it having major applications in science and engineering. In reality, it offers many possibly valuable techniques for resolving differential and integral equations and numerous other concerns that include particular equations, mathematical physics features, as their augmentations and speculations in a single variable, and n is the limit from there. A portion of the regions of current utilization of fractional calculus incorporates rheology, electrical networks, probability and statistics, electrochemistry of corrosion, chemical physics, control theory of dynamical systems, optics and signal processing, etc. As of late, numerous attempts have been dedicated to this subject; a couple of them are accounted for in [1,2,3,4]. The examination of evaluating fractional derivative operators is consistently a hotly debated issue of research. Many attempts have been given lately, and numerous revelations have been made in this course; some of them are recorded in [5,6,7,8,9].

The model which represents the motion of electromagnetic waves is termed the perturbed nonlinear Schrodinger equation (NLSE). The new type of soliton solutions of time-fractional perturbed NLSE with conformable derivative in nonlinear negative-index materials with Bohm potential are discussed in [10]. The Perturbed NLSE with a conformable space–time fractional model is studied in [11]. Now in the present work, we employ the New extended algebraic method to find solitary wave solutions of fractional Perturbed NLSE with beta and M-truncated derivatives. The results are new and not seen in the literature.

The equation of consideration here depicts the elements of soliton propagation through optical meta-materials with self-steepening (SS), interemodal dispersion (IMD), Bohm potential and nonlinear dispersion (ND) and is of the type [12,13,14]

(1) $$\begin{array}{cc} iD_{\tau }^{\mu }v+av_{\xi \xi }+{\left(b|v\right|}^{2})v= & i(\theta_{1}\frac{v}{\left|v\right|}{\left|v\right|}_{\xi \xi }+\theta_{2}v_{\xi \xi }+\theta_{3}{\left(|v\right|}^{2}{v)}_{\xi }+\theta_{4}{\left(|v\right|}^{2}{)}_{\xi }v)+c_{1}{\left(|v\right|}^{2}{v)}_{\xi \xi }\\ & c_{2}{\left|v\right|}^{2}{\left(v\right)}_{\xi \xi }+c_{3}v^{2}{\left(v^{⋆}\right)}_{\xi \xi }. \end{array}$$

The complex function v(ξ,τ) is the dependent variable which shows the wave profile, μ is the fractional parameter with a value between zero and one, ξ indicates the non-dimensional distance across the fiber and the temporal component is τ. Furthermore, coefficient parameter *a* is the group velocity dispersion while *b* describes the cubic nonlinearity known as self-phase modulation.

The paper divided as follows. In Section 1.1 and Section 1.2 main definitions for fractional derivatives are reported. In Section 2, we present governing equations and mathematical analysis of the given equation. In Section 3, we present the fundamentals of the new extended direct algebraic method and use this algorithm to find the DE. In Section 4, we describe necessary and sufficient conditions and physical features of some obtained solutions. The conclusion is stated at the end.

### 1.1. Beta Derivative

**Definition** **1.** *The beta derivative is defined by [8]:*

(2) $${}_{0}^{E}D_{\xi }^{\mu }\left(G\left(\xi \right)\right)=\lim_{\varepsilon \to 0}\frac{G\left(\xi +\varepsilon \left(\xi +\frac{1}{\Gamma (\alpha )}\right)\right)-G\left(\xi \right)}{\varepsilon },$$

*with a few properties as labeled below:*

**Theorem** **1.** *Let 0<μ≤1, δ,λ∈R. f and g are $\mu^{th}$ order differentiable functions at τ>0. Then, we have:*

***1:*** *${}_{0}^{E}D_{\xi }^{\mu }\left(\delta f(\xi )+\lambda g(\xi )\right)=\delta {}_{0}^{E}D_{\xi }^{\mu }\left(f(\xi )\right)+\lambda {}_{0}^{E}D_{\xi }^{\mu }\left(g(\xi )\right)$,*

***2:*** *$D_{\xi }^{\mu }\left(c\right)=0$, here c is constant.*

***3:*** *${}_{0}^{E}D_{\xi }^{\mu }\left(f\left(\xi \right)*g\left(\xi \right)\right)=g\left(\xi \right){}_{0}^{E}D_{\xi }^{\mu }\left(f(\xi )\right)+f\left(\xi \right){}_{0}^{E}D_{\xi }^{\mu }\left(g(\xi )\right)$,*

***4:*** *${}_{0}^{E}D_{\xi }^{\mu }\left(\frac{f(\xi )}{g(\xi )}\right)=\frac{g\left(\xi \right){}_{0}^{E}D_{\xi }^{\mu }f\left(\xi \right)-f\left(\xi \right){}_{0}^{E}D_{\xi }^{\mu }g\left(\xi \right)}{g^{2}\left(\xi \right)}$,*

***5:*** *For $\varepsilon ={\left(\xi +\frac{1}{\Gamma (\mu )}\right)}^{1-\mu }h,h\to 0$ when ε→0; therefore, we have*

$${}_{0}^{E}D_{\xi }^{\mu }f\left(\xi \right)={\left(\xi +\frac{1}{\Gamma (\mu )}\right)}^{1-\mu }\frac{df(\xi )}{d\xi }$$

*with $\xi =\frac{\nu }{\mu }{\left(\xi +\frac{1}{\Gamma (\mu )}\right)}^{\mu }$, where ν is a constant.*

***6:*** *${}_{0}^{E}D_{\xi }^{\mu }\left(\frac{f(\tau )}{g(\xi )}\right)=\tau \frac{df(\tau )}{d\tau }$.*

### 1.2. Truncated M-Fractional Derivative

**Definition** **2.** *The truncated Mittag–Leffler function with one parameter is defined as:*

(3) $${}_{i}T_{\Theta }\left(y\right)=\sum_{k=0}^{i}\frac{y^{k}}{\Gamma (\Theta k+1)},$$

*where Θ>0 *&* y∈C. It is characterized by a non-fuzzy idea as illustrated below.*

**Definition** **3.** *Assume G:[0,∞)→R, and μ∈(0,1) the TMD of G of order μ is given by:*

(4) $${}_{i}D_{M}^{\mu ,\Theta }G\left(\tau \right)=\lim_{\varepsilon \to 0}\frac{G\left(\tau +{}_{i}T_{\Theta }\left(\varepsilon \tau^{-\mu }\right)\right)-G\left(\tau \right)}{\varepsilon },$$

*for τ>0 and ${}_{i}T_{\Theta }\left(.\right)$, Θ>0 is defined in the above definition.*

**Theorem** **2.** *Let μ∈(0,1], Θ>0. G and H are $\mu^{th}$ order differentiable functions at τ>0. Then, we have:*

***1-*** *${}_{i}D_{M}^{\mu ,\Theta }\left(p_{1}G+p_{2}H\right)=p_{1}{}_{i}D_{M}^{\mu ,\Theta }\left(G\right)+p_{2}{}_{i}D_{M}^{\mu ,\Theta }\left(H\right),$ where $p_{1},p_{2}\in R$*

***2-*** *${}_{i}D_{M}^{\mu ,\Theta }\left(\tau^{\sigma }\right)=\sigma \tau^{\sigma -\mu },\sigma \in R$,*

***3-*** *${}_{i}D_{M}^{\mu ,\Theta }\left(GH\right)=G{}_{i}D_{M}^{\mu ,\Theta }\left(H\right)+H{}_{i}D_{M}^{\mu ,\Theta }\left(G\right)$,*

***4-*** *${}_{i}D_{M}^{\mu ,\Theta }\left(\frac{G}{H}\right)=\frac{G{}_{i}D_{M}^{\mu ,\Theta }\left(H\right)-H{}_{i}D_{M}^{\mu ,\Theta }\left(G\right)}{H^{2}},$
*

***5-*** *${}_{i}D_{M}^{\mu ,\Theta }\left(G\right)\left(\tau \right)=\frac{\tau^{1-\mu }}{\Gamma (\Theta +1)}\frac{dG}{d\tau }$,*

***6-*** *${}_{i}D_{M}^{\mu ,\Theta }\left(G\circ H\right)\left(\tau \right)=f^{\prime }\left(H\left(\tau \right)\right){}_{i}D_{M}^{\mu ,\Theta }H\left(\tau \right)$.*

## 2. Governing Equations

By considering a beta derivative, the above equation can be composed as:

(5) $$i{}_{0}^{E}D_{\tau }^{\mu }\left(v\right)+a{}_{0}^{E}D_{\xi }^{2\mu }\left(v\right)+{\left(b|u\right|}^{2}v)=i\left(\theta_{1}\frac{v}{\left|v\right|}{}_{0}^{E}D_{\xi }^{2\mu }\left(|v|\right)+\theta_{2}{}_{0}^{E}D_{\xi }^{\mu }\left(v\right)+\theta_{3}{}_{0}^{E}D_{\xi }^{\mu }{\left(|v\right|}^{2}v)+\theta_{4}{}_{0}^{E}D_{\xi }^{\mu }{\left(|v\right|}^{2})v\right)\;+c_{1}{}_{0}^{E}D_{\xi }^{2\mu }{\left(|v\right|}^{2}v)+c_{2}{\left|v\right|}^{2}{}_{0}^{E}D_{\xi }^{2\mu }\left(v\right)+c_{3}v^{2}{}_{0}^{E}D_{\xi }^{2\mu }\left(v^{⋆}\right),$$

Meanwhile by taking the M-truncated derivative into account, the model under investigation takes the structure as below:

(6) $$\begin{array}{cc} \; & i{}_{0}^{E}D_{M,\tau }^{\mu ,\Theta }\left(v\right)+a{}_{0}^{E}D_{M,\xi }^{2\mu ,\Theta }\left(v\right)+{\left(b|u\right|}^{2}v)=i(\theta_{1}\frac{v}{\left|v\right|}{}_{0}^{E}D_{M,\xi }^{2\mu ,\Theta }\left(|v|\right)+\theta_{2}{}_{0}^{E}D_{M,\xi }^{\mu ,\Theta }\left(v\right)+\theta_{3}{}_{0}^{E}D_{M,\xi }^{\mu ,\Theta }{\left(|v\right|}^{2}v)\\ & \;\;\;\;\;\;\;\;\;\;\;\;\;\;\;\;\;\;\;\;\;\;\;\;\;\;+\theta_{4}{}_{0}^{E}D_{M,\xi }^{\mu ,\Theta }{\left(|v\right|}^{2})v)+c_{1}{}_{0}^{E}D_{M,\xi }^{2\mu ,\Theta }{\left(|v\right|}^{2}v)+c_{2}{\left|v\right|}^{2}{}_{0}^{E}D_{M,\xi }^{2\mu ,\Theta }\left(v\right)+c_{3}v^{2}{}_{0}^{E}D_{M,\xi }^{2\mu ,\Theta }\left(v^{⋆}\right), \end{array}$$

in above ${}_{0}^{E}D_{M,\tau }^{\mu ,\Theta }$ and ${}_{0}^{E}D_{M,\xi }^{\mu ,\Theta }$ are M-truncated derivatives with τ and ξ, respectively.

### Mathematical Survey

For the solution of Equation (1), Equations (5) and (6) the first step is follows:

(7) $$v\left(\xi ,\tau \right)=u\left(\eta \right)e^{i\psi (\xi ,\tau )},$$

where the v(ξ,τ) represents pulse shape of soliton. In sense of the beta derivative, we have

(8) $$\eta =\frac{1}{\mu }{\left(\xi +\frac{1}{\Gamma (\mu )}\right)}^{\mu }-\frac{\nu }{\mu }{\left(\tau +\frac{1}{\Gamma (\mu )}\right)}^{\mu }$$

and

(9) $$\psi \left(\xi ,\tau \right)=-\frac{k}{\mu }{\left(\xi +\frac{1}{\Gamma (\mu )}\right)}^{\mu }+\frac{\omega }{\mu }{\left(\tau +\frac{1}{\Gamma (\mu )}\right)}^{\mu }+\theta_{0}\left(\eta \right).$$

By the virtue of M-truncated derivative, we have:

(10) $$\eta =\frac{\Gamma (\Theta +1)}{\mu }\left(\xi^{\mu }-\nu \tau^{\mu }\right),$$

(11) $$\psi \left(\xi ,\tau \right)=-\frac{\Gamma (\Theta +1)}{\mu }\left(\kappa \xi^{\mu }-\nu \omega \tau^{\mu }\right)+\theta_{0}\left(\eta \right),$$

where ψ(ξ,τ), κ, ω, ν and $\theta_{0}\left(\eta \right)$ are the phase component, frequency, wave number, speed and phase function of soliton, respectively. Putting these values in Equation (1), Equations (5) and (6), then the imaginary part results in:

(12) $$\nu =\theta_{1}-2a\kappa$$

and

(13) $$3\theta_{2}+2\theta_{4}-2\kappa \left(3c_{1}+c_{2}-c_{3}\right)=0,$$

while the real part implies

(14) $$au^{\prime \prime }-\left(\omega +a\kappa^{2}+\kappa \theta_{2}\right)u+\left(b-\kappa \theta_{3}+\kappa^{2}c_{1}+\kappa^{2}c_{2}+\kappa^{2}c_{3}\right)u^{3}-\left(3c_{1}+c_{2}+c_{3}\right)u^{2}u^{\prime \prime }-6c_{1}u^{\prime^{2}}=0.$$

We apply the accompanying changes $c_{1}=0$, and $c_{2}=-c_{3}$ in Equation (14) for its solution as

(15) $$au^{\prime \prime }-\left(\omega +a\kappa^{2}+\theta_{2}\kappa \right)u+\left(b-\kappa \theta_{3}\right)u^{3}=0,$$

where

(16) $$3\theta_{2}+2\theta_{4}+4\kappa c_{3}=0.$$

## 3. Applications

This section is devoted to the application of the method with two different definitions of the derivatives.

### 3.1. The New Extended Direct Algebraic Method

In this section, the general procedure of this method [15,16,17] is reported in two steps as given below:

**Step: 1**  Suppose that the given nonlinear PDE is of the form:
  (17) $$G(v,v_{\xi },v_{\tau },v_{\xi \xi },v_{\tau \tau },...)=0,$$
  where *v* represents the dependent variable and ξ, τ are the independent variables.

By using the wave transformation:

v(ξ,τ)=u(η),η=ξ−cτ.

Equation (17) can be transformed into nonlinear ODE:

(18) $$J(u,u^{\prime },u^{\prime \prime },u^{\prime \prime \prime }...)=0.$$

**Step: 2**  We assume the solution of the ODE (18) of the type:
  (19) $$u\left(\eta \right)=\sum_{i=0}^{m}b_{i}Z^{i}\left(\eta \right),$$
  where $b_{i}$(0<i≤n) are the coefficients and Z(η) satisfies the ODE of the type:
  (20) $$Z^{\prime }\left(\eta \right)=\ln\left(B\right)\left(\alpha +\beta Z\left(\eta \right)+\gamma Z^{2}\left(\eta \right)\right),\;B\neq 0,1,$$
  where α, β and γ are the constants. The Equation (20) has the solution written as:
  1: When $\beta^{2}-4\alpha \gamma <0$ and γ≠0,
  $Z_{1}\left(\eta \right)=-\frac{\beta }{2\gamma }+\frac{\sqrt{-(\beta^{2}-4\alpha \gamma )}}{2\gamma }\tan_{B}\left(\frac{\sqrt{-(\beta^{2}-4\alpha \gamma )}}{2}\eta \;\right)$,
  $Z_{2}\left(\eta \right)=-\frac{\beta }{2\gamma }-\frac{\sqrt{-(\beta^{2}-4\alpha \gamma )}}{2\gamma }\cot_{B}\left(\frac{\sqrt{-(\beta^{2}-4\alpha \gamma )}}{2}\eta \;\right)$,
  $Z_{3}\left(\eta \right)=-\frac{\beta }{2\gamma }+\frac{\sqrt{-(\beta^{2}-4\alpha \gamma )}}{2\gamma }\left(\tan_{B}\left(\sqrt{-(\beta^{2}-4\alpha \gamma )}\;\eta \;\right)\pm \sqrt{mn}\sec_{B}\left(\sqrt{-(\beta^{2}-4\alpha \gamma )}\;\eta \;\right)\right)$,
  $Z_{4}\left(\eta \right)=-\frac{\beta }{2\gamma }-\frac{\sqrt{-(\beta^{2}-4\alpha \gamma )}}{2\gamma }\left(\cot_{B}\left(\sqrt{-(\beta^{2}-4\alpha \gamma )}\;\eta \;\right)\pm \sqrt{mn}\csc_{B}\left(\sqrt{-(\beta^{2}-4\alpha \gamma )}\;\eta \;\right)\right)$,
  $Z_{5}\left(\eta \right)=-\frac{\beta }{2\gamma }+\frac{\sqrt{-(\beta^{2}-4\alpha \gamma )}}{4\gamma }\left(\tan_{B}\left(\frac{\sqrt{-(\beta^{2}-4\alpha \gamma )}}{4}\;\eta \;\right)-\cot_{B}\left(\frac{\sqrt{-(\beta^{2}-4\alpha \gamma )}}{4}\;\eta \;\right)\right)$.
  2: When $\beta^{2}-4\alpha \gamma >0$ and γ≠0,
  $Z_{6}\left(\eta \right)=-\frac{\beta }{2\gamma }-\frac{\sqrt{\beta^{2}-4\alpha \gamma }}{2\gamma }\tan_{B}\left(\frac{\sqrt{\beta^{2}-4\alpha \gamma }}{2}\eta \;\right)$,
  $Z_{7}\left(\eta \right)=-\frac{\beta }{2\gamma }-\frac{\sqrt{\beta^{2}-4\alpha \gamma }}{2\gamma }\cot_{B}\left(\frac{\sqrt{\beta^{2}-4\alpha \gamma }}{2}\eta \;\right)$,
  $Z_{8}\left(\eta \right)=-\frac{\beta }{2\gamma }-\frac{\sqrt{\beta^{2}-4\alpha \gamma }}{2\gamma }\left(\tanh_{B}\left(\sqrt{\beta^{2}-4\alpha \gamma }\;\eta \;\right)\pm \iota \sqrt{mn}\mathrm{sech}{\;}_{B}\left(\sqrt{\beta^{2}-4\alpha \gamma }\;\eta \;\right)\right)$,
  $Z_{9}\left(\eta \right)=-\frac{\beta }{2\gamma }-\frac{\sqrt{\beta^{2}-4\alpha \gamma }}{2\gamma }\left(\coth_{B}\left(\sqrt{\beta^{2}-4\alpha \gamma }\;\eta \;\right)\pm \sqrt{mn}\mathrm{csch}{\;}_{B}\left(\sqrt{\beta^{2}-4\alpha \gamma }\;\eta \;\right)\right)$,
  $Z_{10}\left(\eta \right)=-\frac{\beta }{2\gamma }-\frac{\sqrt{\beta^{2}-4\alpha \gamma }}{4\gamma }\left(\tanh_{B}\left(\frac{\sqrt{\beta^{2}-4\alpha \gamma }}{4}\;\eta \;\right)+\coth_{B}\left(\frac{\sqrt{\beta^{2}-4\alpha \gamma }}{4}\;\eta \;\right)\right)$.
  3: When αγ>0 and β=0,
  $Z_{11}\left(\eta \right)=\sqrt{\frac{\alpha }{\gamma }}\tan_{B}\left(\sqrt{\alpha \gamma }\;\eta \right)$,
  $Z_{12}\left(\eta \right)=-\sqrt{\frac{\alpha }{\gamma }}\cot_{B}\left(\sqrt{\alpha \gamma }\;\eta \right)$,
  $Z_{13}\left(\eta \right)=\sqrt{\frac{\alpha }{\gamma }}\left(\tan_{B}\left(2\sqrt{\alpha \gamma }\;\eta \right)\pm \sqrt{mn}\sec_{B}\left(2\sqrt{\alpha \gamma }\;\eta \right)\right)$,
  $Z_{14}\left(\eta \right)=-\left(\sqrt{\frac{\alpha }{\gamma }}(\cot_{B}\left(2\sqrt{\alpha \gamma }\;\eta \right)\pm \sqrt{mn}\csc_{B}\left(2\sqrt{\alpha \gamma }\;\eta \right)\right)$,
  $Z_{15}\left(\eta \right)=\frac{1}{2}\sqrt{\frac{\alpha }{\gamma }}\left(\tan_{B}\left(\frac{\sqrt{\alpha \gamma }}{2}\;\eta \right)-\cot_{B}\left(\frac{\sqrt{\alpha \gamma }}{2}\;\eta \right)\right)$.
  4: When αγ<0 and β=0,
  $Z_{16}\left(\eta \right)=-\sqrt{-\frac{\alpha }{\gamma }}\tanh_{B}\left(\sqrt{-\alpha \gamma }\;\eta \right)$,
  $Z_{17}\left(\eta \right)=-\sqrt{-\frac{\alpha }{\gamma }}\coth_{B}\left(-\sqrt{\alpha \gamma }\;\eta \right)$,
  $Z_{18}\left(\eta \right)=-\sqrt{\frac{-\alpha }{\gamma }}\left(\tanh_{B}\left(2\sqrt{-\alpha \gamma }\;\eta \right)\pm \iota \sqrt{mn}\mathrm{sech}{\;}_{B}\left(2\sqrt{-\alpha \gamma }\;\eta \right)\right)$,
  $Z_{19}\left(\eta \right)=-\sqrt{-\frac{\alpha }{\gamma }}\left(\coth_{B}\left(2\sqrt{-\alpha \gamma }\;\eta \right)\pm \sqrt{mn}\mathrm{csch}{\;}_{B}\left(2\sqrt{-\alpha \gamma }\;\eta \right)\right)$,
  $Z_{20}\left(\eta \right)=-\frac{1}{2}\sqrt{\frac{-\alpha }{\gamma }}\left(\tanh_{B}\left(\frac{\sqrt{-\alpha \gamma }}{2}\;\eta \right)+\coth_{B}\left(\frac{\sqrt{-\alpha \gamma }}{2}\;\eta \right)\right)$.
  5: When β=0 and γ=α,
  $Z_{21}\left(\eta \right)=\tan_{B}\left(\alpha \eta \right)$,
  $Z_{22}\left(\eta \right)=-\cot_{B}\left(\alpha \eta \right)$,
  $Z_{23}\left(\eta \right)=\tan_{B}\left(2\alpha \eta \right)\pm \sqrt{mn}\sec_{B}\left(2\alpha \eta \right)$,
  $Z_{24}\left(\eta \right)=-\cot_{B}\left(2\alpha \eta \right)\pm \sqrt{mn}\csc_{B}\left(2\alpha \eta \right)$,
  $Z_{25}\left(\eta \right)=\frac{1}{2}\left(\tan_{B}\left(\frac{\alpha }{2}\eta \right)-\cot_{B}\left(\frac{\alpha }{2}\eta \right)\right)$.
  6: When β=0 and γ=−α,
  $Z_{26}\left(\eta \right)=-\tanh_{B}\left(\alpha \eta \right)$,
  $Z_{27}\left(\eta \right)=-\coth_{B}\left(\alpha \eta \right)$,
  $Z_{28}\left(\eta \right)=-\tanh_{B}\left(2\alpha \eta \right)\pm \iota \sqrt{mn}\mathrm{sech}{\;}_{B}\left(2\alpha \eta \right)$,
  $Z_{29}\left(\eta \right)=-\coth_{B}\left(2\alpha \eta \right)\pm \sqrt{mn}\mathrm{csch}{\;}_{B}\left(2\alpha \eta \right)$,
  $Z_{30}\left(\eta \right)=-\frac{1}{2}\left(\tanh_{B}\left(\frac{\alpha }{2}\eta \right)+\coth_{B}\left(\frac{\alpha }{2}\eta \right)\right)$.
  7: When $\beta^{2}=4\alpha \gamma$,
  $Z_{31}\left(\eta \right)=\frac{-2\alpha (\beta \eta \ln(B)+2)}{\beta^{2}\eta \ln\left(B\right)}$.
  8: When β=ρ, α=qρ(q≠0) and γ=0,
  $Z_{32}\left(\eta \right)=B^{\rho \eta }-q$.
  9: When β=γ=0,
  $Z_{33}\left(\eta \right)=\alpha \eta \ln\left(B\right)$.
  10: When β=α=0,
  $Z_{34}\left(\eta \right)=\frac{-1}{\gamma \eta \ln(B)}$.
  11: When α=0 and β≠0,
  $Z_{35}\left(\eta \right)=-\frac{s\beta }{\gamma (\cosh_{B}\left(\beta \eta \right)-\sinh_{B}\left(\beta \eta \right)+s)}$,
  $Z_{36}\left(\eta \right)=-\frac{\beta (\sinh_{B}\left(\beta \eta \right)+\cosh_{B}\left(\beta \eta \right))}{\gamma (\sinh_{B}\left(\beta \eta \right)+\cosh_{B}\left(\beta \eta \right)+r)}$.
  12: When β=ρ, γ=mρ(m≠0) and α=0,
  $Z_{37}\left(\eta \right)=\frac{sB^{\rho \eta }}{r-msB^{\rho \eta }}$.

Now, the hyperbolic and trigonometric functions are given as follows:

$\sinh_{B}\left(\eta \right)=\frac{qB^{\eta }-sB^{-\eta }}{2}$, $\cosh_{B}\left(\eta \right)=\frac{rB^{\eta }+sB^{-\eta }}{2}$,

$\tanh_{B}\left(\eta \right)=\frac{rB^{\eta }-sB^{-\eta }}{rB^{\eta }+sB^{-\eta }}$, $\coth_{B}\left(\eta \right)=\frac{rB^{\eta }+sB^{-\eta }}{rB^{\eta }-sB^{-\eta }}$,

$\mathrm{csch}{\;}_{B}\left(\eta \right)=\frac{2}{rB^{\eta }-sB^{-\eta }}$, $\mathrm{sech}{\;}_{B}\left(\eta \right)=\frac{2}{rB^{\eta }+sB^{-\eta }}$,

$\sin_{B}\left(\eta \right)=\frac{rB^{\iota \eta }-sB^{-\iota \eta }}{2\iota }$, $\cos_{B}\left(\eta \right)=\frac{rB^{\iota \eta }+sB^{-\iota \eta }}{2}$,

$\tan_{B}\left(\eta \right)=-i\frac{rB^{\iota \eta }-sB^{-\iota \eta }}{rB^{\iota \eta }+sB^{-\iota \eta }}$, $\cot_{B}\left(\eta \right)=i\frac{rB^{\iota \eta }+sB^{-\iota \eta }}{rB^{\iota \eta }-sB^{-\iota \eta }}$,

$\csc_{B}\left(\eta \right)=\frac{2\iota }{rB^{\iota \eta }-sB^{-\iota \eta }}$, $\mathrm{sech}{\;}_{B}\left(\eta \right)=\frac{2}{rB^{\iota \eta }+sB^{-\iota \eta }}$,

where *r* and *s* are constants which are known as the deformation parameters.

### 3.2. Application to the NLS Equation

Let us take the transformation of the form:

(21) $$v\left(\xi ,\tau \right)=u\left(\eta \right)e^{i\psi (\xi ,\tau )},$$

by using the balancing scheme on these terms $u^{3}$ and $u^{\prime \prime }$ of Equation (15), assigns m=1 to Equation (19). We obtain the following transformation:

(22) $$u\left(\eta \right)=b_{0}+b_{1}Z\left(\eta \right),$$

putting Equation (22) into (15), then collecting the coefficients of different powers of Z(η), we obtain a system of the following algebraic equations:

(23) $$\begin{array}{c} \begin{array}{cc} Z^{3}\left(\eta \right) & :2ab_{1}\gamma^{2}\ln{\left(B\right)}^{2}-b_{1}^{3}\kappa \theta_{3}+b_{1}^{3}b=0,\\ Z^{2}\left(\eta \right) & :3ab_{1}\beta \ln{\left(B\right)}^{2}\gamma -3b_{0}b_{1}^{2}\kappa \theta_{3}+3b_{0}b_{1}^{2}b=0,\\ Z^{1}\left(\eta \right) & :ab_{1}\beta^{2}\ln{\left(B\right)}^{2}+2ab_{1}\gamma \ln{\left(B\right)}^{2}\alpha -b_{1}a\kappa^{2}-b_{1}\theta_{2}\kappa -b_{1}\omega -3b_{0}^{2}b_{1}\kappa \theta_{3}+3b_{0}^{2}b_{1}b=0,\\ Z^{0}\left(\eta \right) & :ab_{1}\beta \ln{\left(B\right)}^{2}\alpha -b_{0}a\kappa^{2}-b_{0}\theta_{2}\kappa -b_{0}\omega -b_{0}^{3}\kappa \theta_{3}+b_{0}^{3}b=0. \end{array} \end{array}$$

The solution of the system of Equation (23) by use of Maple for $b_{0}$, $b_{1}$ and ω, we obtain the following values:

(24) $$\begin{array}{c} \begin{array}{cc} b_{0} & =\pm \frac{\gamma a\beta \ln(B)}{\sqrt{-\frac{2\gamma^{2}a}{b-\kappa \theta_{3}}}\left(b-\kappa \theta_{3}\right)},\;\;b_{1}=\pm \sqrt{-\frac{2\gamma^{2}a}{b-\kappa \theta_{3}}}\ln\left(B\right),\\ \omega & =2\gamma \ln{\left(B\right)}^{2}a\alpha -\frac{1}{2}\ln{\left(B\right)}^{2}a\beta^{2}-a\kappa^{2}-\kappa \theta_{2}. \end{array} \end{array}$$

Let us consider $\Delta =\beta^{2}-4\alpha \gamma$ and $\Pi =\gamma \sqrt{-\frac{2a}{b-\kappa \theta_{3}}}$.

Then $b_{0}=\pm \frac{a\beta \ln(B)}{\Pi (b-\kappa \theta_{3})}$ and $b_{1}=\pm \Pi \ln\left(B\right)$.

From Equations (21), (22) and (24) and the different cases of solutions of Equation (20), we obtain the solutions which come after:

Case1. When Δ<0 and γ≠0,

$v_{1}\left(\xi ,\tau \right)=\pm \frac{a\ln(B)}{\Pi (b-\kappa \theta_{3})}\left(\sqrt{-\Delta }\tan_{B}\left(\frac{\sqrt{-\Delta }}{2}\eta \right)\right)e^{i\psi (\xi ,\tau )}$,

$v_{2}\left(\xi ,\tau \right)=\pm \frac{a\ln(B)}{\Pi (b-\kappa \theta_{3})}\left(\sqrt{-\Delta }\cot_{B}\left(\frac{\sqrt{-\Delta }}{2}\eta \right)\right)e^{i\psi (\xi ,\tau )}$,

$v_{3}\left(\xi ,\tau \right)=\pm \frac{a\ln(B)}{\Pi (b-\kappa \theta_{3})}\left(2\beta -\sqrt{-\Delta }\left(\tan_{B}\left(\sqrt{-\Delta }\eta \right)\pm \sqrt{mn}\sec_{B}\left(\sqrt{-\Delta }\eta \right)\right)\right)e^{i\psi (\xi ,\tau )}$,

$v_{4}\left(\xi ,\tau \right)=\pm \frac{a\ln(B)}{\Pi (b-\kappa \theta_{3})}\left(2\beta -\sqrt{-\Delta }\left(-\cot_{B}\left(\sqrt{-\Delta }\eta \right)\pm \sqrt{mn}\csc_{B}\left(\sqrt{-\Delta }\eta \right)\right)\right)e^{i\psi (\xi ,\tau )}$,

$v_{5}\left(\xi ,\tau \right)=\pm \frac{a\ln(B)}{\Pi (b-\kappa \theta_{3})}\left(2\beta -\sqrt{-\Delta }(\tan_{B}\left(\frac{\sqrt{-\Delta }}{4}\eta \right)\pm \cot_{B}\left(\frac{\sqrt{-\Delta }}{4}\eta \right)\right))e^{i\psi (\xi ,\tau )}$.

Case2. When Δ>0 and γ≠0,

$v_{6}\left(\xi ,\tau \right)=\pm \frac{a\ln(B)}{\Pi (b-\kappa \theta_{3})}\left(\sqrt{\Delta }\tanh_{B}\left(\frac{\sqrt{\Delta }}{2}\eta \right)\right)e^{i\psi (\xi ,\tau )}$,

$v_{7}\left(\xi ,\tau \right)=\pm \frac{a\ln(B)}{\Pi (b-\kappa \theta_{3})}\left(\sqrt{\Delta }\coth_{B}\left(\frac{\sqrt{\Delta }}{2}\eta \right)\right)e^{i\psi (\xi ,\tau )}$,

$v_{8}\left(\xi ,\tau \right)=\pm \frac{a\ln(B)}{\Pi (b-\kappa \theta_{3})}\left(2\beta -\sqrt{\Delta }\left(-\tanh_{B}\left(\sqrt{\Delta }\eta \right)\pm i\sqrt{mn}\;\mathrm{sech}{\;}_{B}\left(\sqrt{\Delta }\eta \right)\right)\right)e^{i\psi (\xi ,\tau )}$,

$v_{9}\left(\xi ,\tau \right)=\pm \frac{a\ln(B)}{\Pi (b-\kappa \theta_{3})}\left(2\beta -\sqrt{\Delta }\left(-\coth_{B}\left(\sqrt{\Delta }\eta \right)\pm \sqrt{mn}\;\mathrm{csch}{\;}_{B}\left(\sqrt{\Delta }\eta \right)\right)\right)e^{i\psi (\xi ,\tau )}$,

$v_{10}\left(\xi ,\tau \right)=\pm \frac{a\ln(B)}{\Pi (b-\kappa \theta_{3})}\left(2\beta -\sqrt{\Delta }(-\tanh_{B}\left(\frac{\sqrt{\Delta }}{4}\eta \right)\pm \coth_{B}\left(\frac{\sqrt{\Delta }}{4}\eta \right)\right))e^{i\psi (\xi ,\tau )}$.

Case3. When γα>0 and β=0,

$v_{11}\left(\xi ,\tau \right)=\pm \Pi \ln\left(B\right)\sqrt{\alpha \gamma }\tan_{B}\left(\sqrt{\alpha \gamma }\eta \right)e^{i\psi (\xi ,\tau )}$,

$v_{12}\left(\xi ,\tau \right)=\pm \Pi \ln\left(B\right)\sqrt{\alpha \gamma }\cot_{B}\left(\sqrt{\alpha \gamma }\eta \right)e^{i\psi (\xi ,\tau )}$,

$v_{13}\left(\xi ,\tau \right)=\pm \Pi \ln\left(B\right)\sqrt{\alpha \gamma }\left(\tan_{B}\left(2\sqrt{\alpha \gamma }\eta \right)\pm \sqrt{mn}\;\sec_{B}\left(2\sqrt{\alpha \gamma }\eta \right)\right)e^{i\psi (\xi ,\tau )}$,

$v_{14}\left(\xi ,\tau \right)=\pm \Pi \ln\left(B\right)\sqrt{\alpha \gamma }\left(-\cot_{B}\left(2\sqrt{\alpha \gamma }\eta \right)\pm \sqrt{mn}\;\csc_{B}\left(2\sqrt{\alpha \gamma }\eta \right)\right)e^{i\psi (\xi ,\tau )}$,

$v_{15}\left(\xi ,\tau \right)=\pm \frac{1}{2}\Pi \ln\left(B\right)\sqrt{\alpha \gamma }\left(\tan_{B}\left(\frac{\sqrt{\alpha \gamma }}{2}\eta \right)\pm \cot_{B}\left(\frac{\sqrt{\alpha \gamma }}{2}\eta \right)\right)e^{i\psi (\xi ,\tau )}$.

Case4. When γα<0 and β=0,

$v_{16}\left(\xi ,\tau \right)=\pm \Pi \ln\left(B\right)\sqrt{-\alpha \gamma }\tanh_{B}\left(\sqrt{-\alpha \gamma }\eta \right)e^{i\psi (\xi ,\tau )}$,

$v_{17}\left(\xi ,\tau \right)=\pm \Pi \ln\left(B\right)\sqrt{-\alpha \gamma }\coth_{B}\left(\sqrt{-\alpha \gamma }\eta \right)e^{i\psi (\xi ,\tau )}$,

$v_{18}\left(\xi ,\tau \right)=\pm \Pi \ln\left(B\right)\sqrt{-\alpha \gamma }\left(-\tanh_{B}\left(2\sqrt{-\alpha \gamma }\eta \right)\pm i\sqrt{mn}\;\mathrm{sech}{\;}_{B}\left(2\sqrt{-\alpha \gamma }\eta \right)\right)e^{i\psi (\xi ,\tau )}$,

$v_{19}\left(\xi ,\tau \right)=\pm \Pi \ln\left(B\right)\sqrt{-\alpha \gamma }\left(-\coth_{B}\left(2\sqrt{-\alpha \gamma }\eta \right)\pm \sqrt{mn}\;\mathrm{csch}{\;}_{B}\left(2\sqrt{-\alpha \gamma }\eta \right)\right)e^{i\psi (\xi ,\tau )}$,

$v_{20}\left(\xi ,\tau \right)=\pm \frac{1}{2}\Pi \ln\left(B\right)\sqrt{-\alpha \gamma }\left(-\tanh_{B}\left(\frac{\sqrt{-\alpha \gamma }}{2}\eta \right)\pm \coth_{B}\left(\frac{\sqrt{-\alpha \gamma }}{2}\eta \right)\right)e^{i\psi (\xi ,\tau )}$.

Case5. When β=0 and γ=α,

$v_{21}\left(\xi ,\tau \right)=\pm \Pi \ln\left(B\right)\alpha \tan_{B}\left(\alpha \eta \right)e^{i\psi (\xi ,\tau )}$,

$v_{22}\left(\xi \tau \right)=\pm \Pi \ln\left(B\right)\alpha \cot_{B}\left(\alpha \eta \right)e^{i\psi (\xi ,\tau )}$,

$v_{23}\left(\xi ,\tau \right)=\pm \Pi \ln\left(B\right)\alpha \left(\tan_{B}\left(2\alpha \eta \right)\pm \sqrt{mn}\sec_{B}\left(2\alpha \eta \right)\right)e^{i\psi (\xi ,\tau )}$,

$v_{24}\left(\xi ,\tau \right)=\pm \Pi \ln\left(B\right)\alpha \left(-\cot_{B}\left(2\alpha \eta \right)\pm \sqrt{mn}\csc_{B}\left(2\alpha \eta \right)\right)e^{i\psi (\xi ,\tau )}$,

$v_{25}\left(\xi ,\tau \right)=\pm \frac{1}{2}\Pi \ln\left(B\right)\alpha \left(\tan_{B}\left(\frac{\alpha }{2}\eta \right)\pm \cot_{B}\left(\frac{\alpha }{2}\eta \right)\right)e^{i\psi (\xi ,\tau )}$.

Case6. When β=0 and γ=−α,

$v_{26}\left(\xi ,\tau \right)=\pm \Pi \ln\left(B\right)\alpha \tanh_{B}\left(\alpha \eta \right)e^{i\psi (\xi ,\tau )}$,

$v_{27}\left(\xi \tau \right)=\pm \Pi \ln\left(B\right)\alpha \coth_{B}\left(\alpha \eta \right)e^{i\psi (\xi ,\tau )}$,

$v_{28}\left(\xi ,\tau \right)=\pm \Pi \ln\left(B\right)\alpha (-\tanh_{B}\left(2\alpha \eta \right)\pm i\sqrt{mn}\mathrm{sech}{\;}_{B}\left(2\alpha \eta \right)e^{i\psi (\xi ,\tau )}$,

$v_{29}\left(\xi ,\tau \right)=\pm \Pi \ln\left(B\right)\alpha \left(-\coth_{B}\left(2\alpha \eta \right)\pm \sqrt{mn}\mathrm{csch}{\;}_{B}\left(2\alpha \eta \right)\right)e^{i\psi (\xi ,\tau )}$,

$v_{30}\left(\xi ,\tau \right)=\pm \frac{1}{2}\Pi \ln\left(B\right)\alpha \left(-\tanh_{B}\left(\frac{\alpha }{2}\eta \right)\pm \coth_{B}\left(\frac{\alpha }{2}\eta \right)\right)e^{i\psi (\xi ,\tau )}$.

Case7. When $\beta^{2}=4\alpha \gamma$,

$v_{31}\left(\xi ,\tau \right)=\pm \left(\frac{\alpha \sqrt{4\alpha \gamma }\ln\left(B\right)}{\Pi (b-\kappa \theta_{3})}+\frac{\Pi }{2\alpha \gamma \eta }\left(1+\sqrt{\alpha \gamma }\ln\left(B\right)\eta \right)\right)e^{i\psi (\xi ,\tau )}$,

Case8. When β=ρ, α=qρ(q≠0) and γ=0,

$v_{32}\left(\xi ,\tau \right)=0$.

Case9. When β=γ=0,

$v_{33}\left(\xi ,\tau \right)=0$.

Case10. When β=α=0,

$v_{34}\left(\xi ,\tau \right)=\pm \frac{\Pi }{\gamma \eta }$.

Case11. When α=0 and β≠0,

$v_{35}\left(\xi ,\tau \right)=\pm \Pi \ln\left(B\right)\frac{s\beta }{\gamma \left(\cosh_{B}\left(\beta \eta \right)-\sinh_{B}\left(\beta \eta \right)+s\right)}e^{i\psi (\xi ,\tau )}$,

$v_{36}\left(\xi ,\tau \right)=\pm \Pi \ln\left(B\right)\frac{\beta \left(\sinh_{B}\left(\beta \eta \right)+\cosh_{B}\left(\beta \eta \right)\right)}{\gamma \left(\sinh_{B}\left(\beta \eta \right)+\cosh_{B}\left(\beta \eta \right)+r\right)}e^{i\psi (\xi ,\tau )}$.

Case12. When β=ρ, γ=mρ(m≠0) and α=0,

$v_{37}\left(\xi ,\tau \right)=\pm \Pi \ln\left(B\right)\frac{sB^{\rho \eta }}{r-msB^{\rho \eta }}e^{i\psi (\xi ,\tau )}$,

where η and ψ are given by Equations (8) and (9) for the beta derivative and Equations (10) and (11) for M-truncated derivative.

## 4. Comparison and Discussion

**Remark:** The constraint conditions for the presence of the acquired solutions are given in type as below.

**Proposition:** If v(ξ,τ) is the solitary wave solution of the considered Equation (1), then the conditions for the presence of its solutions are a>0 and $b-\kappa \theta_{3}<0$.

**Remark:** It is essential to mention that the acquired structures of the model under study (Equation (1)) speak to the various sorts of soliton solutions. As $v_{6}$, $v_{16}$, and $v_{26}$ represent dark soliton solutions, $v_{8}$, $v_{18}$, and $v_{28}$ are the dark–bright soliton solutions, $v_{10}$, $v_{20}$, and $v_{30}$ show the dark–singular solutions, $v_{9}$, $v_{19}$, and $v_{29}$ represent a family of singular solutions of type 1 and 2, while $v_{7}$, $v_{17}$, and $v_{27}$ are reported as a singular solution of type 2.

**Figure 1:**
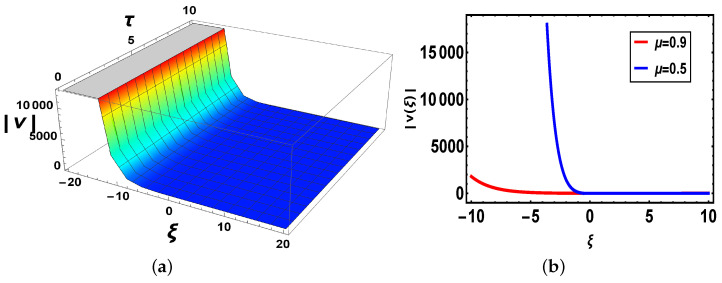
(**a**) 3D-plot with parameter values B=e, a=2.5, μ=0.9, ω=1, b=1, κ=2.3, α=1.2, β=6, γ=2.9, $\theta_{3}=2.7$, ν=4, m=1, n=1 (**b**) 2D-plot for different values of μ of $v_{3}\left(\xi ,\tau \right)$ with beta-derivative.

**Figure 2:**
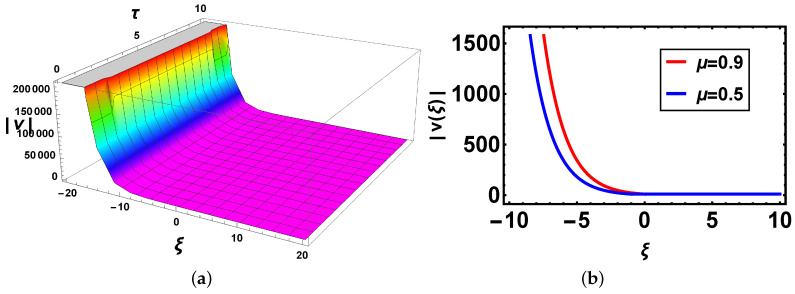
(**a**) 3D and (**b**) 2D plots with the same parameter values as above μ=0.9 and Θ=1.1 of $v_{3}\left(\xi ,\tau \right)$ with the M-truncated derivative.

**Figure 3:**
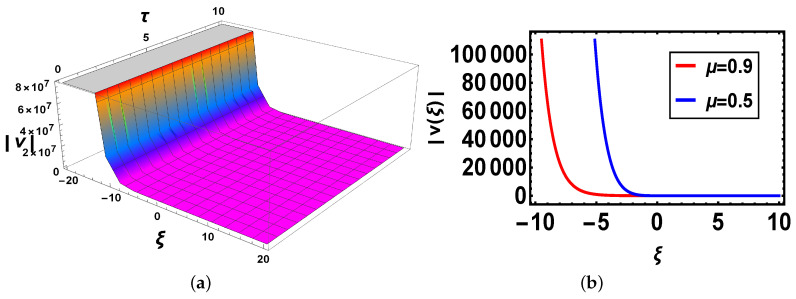
(**a**) 3D and (**b**) 2D plots with same parameter values as above μ=0.9 and Θ=1.1 of $v_{1}\left(\xi ,\tau \right)$ with the beta-derivative.

**Figure 4:**
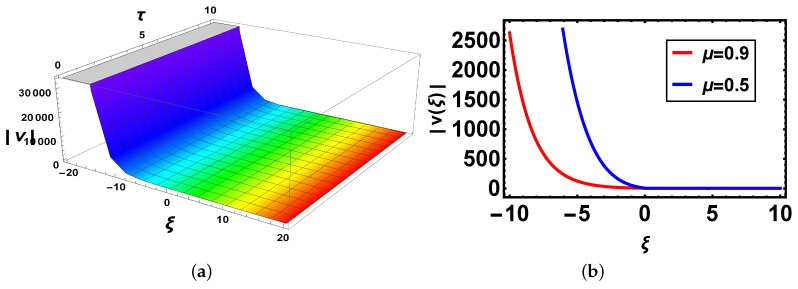
(**a**) 3D and (**b**) 2D plots with same parameter values as above μ=0.9 and Θ=1.1 of $v_{1}\left(\xi ,\tau \right)$ with the M-truncated derivative.

**Figure 5:**
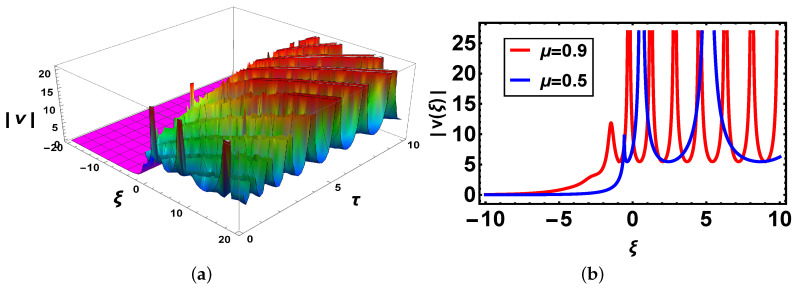
(**a**) 3D and (**b**) 2D plots with same parameter values as above μ=0.5 of $v_{25}\left(\xi ,\tau \right)$ with the beta-derivative.

**Figure 6:**
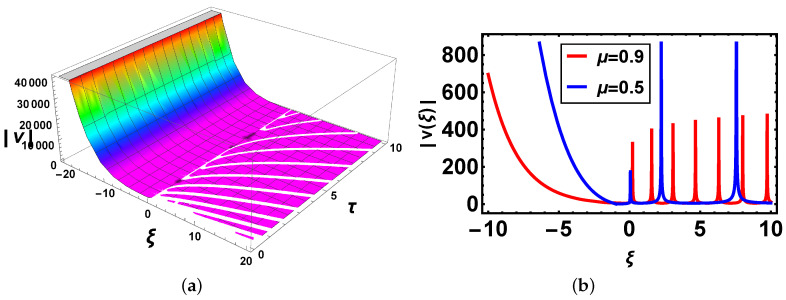
(**a**) 3D and (**b**) 2D plots with same parameter values as above μ=0.5 and Θ=1.1 of $v_{25}\left(\xi ,\tau \right)$ with the M-truncated derivative.

**Comparison:** Now, different solutions are taken into account in the sense of beta and M-truncated derivatives and are depicted in Figure 1, Figure 2, Figure 3, Figure 4, Figure 5 and Figure 6 with various μ’s values.

Figure 1: Figure 1a shows 3D-graph with beta derivative definition, the second indicates a 2D-structure of $v_{3}\left(\xi ,\tau \right)$ when τ=1 by applying two various definitions. It is noted that when τ=1 both definitions show various graphs and overlapping exits in the definite range of the values of the independent variable ξ.

Figure 2: In this figure, the first graph represents its 3D graph for the M-truncated derivative, while Figure 2b shows its structures with the same definition of the derivative but for various values of the fractional parameter μ and τ=1. Here, it is intriguing to take note that the curves have the same structure, and overlapping exists in the definite range of the values of the independent variable ξ similar to the case forgiven in Figure 1b.

Figure 3: In Figure 3a there is 3D graph for the beta derivative for $v_{1}\left(\xi ,\tau \right)$ and Figure 3b shows its structure with the beta definition at τ=1; a 2D structure is seen by utilizing two definitions. It is intriguing to take note that now curves have the same shape and overlapping also exists. In Figure 4, both 3D and 2D structures show the same behavior as in Figure 3. In Figure 5, Figure 5a indicates 3D and Figure 5b shows 2D structures for the beta derivative. What is mentioning here is that the soliton’s amplitude increases for an increase in the value of μ. In Figure 6, represents 3D and 2D graphs, respectively, for the M-truncated derivative. What is also worth mentioning here is that the soliton’s amplitude decreases for an increase in the value of μ.

## 5. Conclusions

In the present work, Equation (1), which describes the propagation of waves in negative-index metamaterials with the Bohm potential term, is taken in the sense of the beta and M-truncated derivatives. The Bohm potential term is accounted for the quantum phase behavior in the NLSE. This exploration elaborates on new families of solitons in negative optical metamaterials. To obtain the various type of solutions, the new extended algebraic method is considered. The considered technique also yielded new families including dark–bright, dark, dark–singular, and singular solutions of Type 1 and 2 of the governing equations. The consequences of this paper are of incredible interest in the fiber optic industry and designed by and large.

## Data Availability

The data that support the findings of this study are available within the article.
